# Phonatory and articulatory representations of speech production in cortical and subcortical fMRI responses

**DOI:** 10.1038/s41598-020-61435-y

**Published:** 2020-03-11

**Authors:** Joao M. Correia, César Caballero-Gaudes, Sara Guediche, Manuel Carreiras

**Affiliations:** 1 BCBL, Basque Center on Cognition Brain and Language, San Sebastian, Spain; 2 Centre for Biomedical Research (CBMR)/Department of Psychology, University of Algarve, Faro, Portugal; 3 Ikerbasque. Basque Foundation for Science, Bilbao, Spain; 4 University of the Basque Country. UPV/EHU, Bilbao, Spain

**Keywords:** Language, Motor cortex

## Abstract

Speaking involves coordination of multiple neuromotor systems, including respiration, phonation and articulation. Developing non-invasive imaging methods to study how the brain controls these systems is critical for understanding the neurobiology of speech production. Recent models and animal research suggest that regions beyond the primary motor cortex (M1) help orchestrate the neuromotor control needed for speaking, including cortical and sub-cortical regions. Using contrasts between speech conditions with controlled respiratory behavior, this fMRI study investigates articulatory gestures involving the tongue, lips and velum (i.e., alveolars versus bilabials, and nasals versus orals), and phonatory gestures (i.e., voiced versus whispered speech). Multivariate pattern analysis (MVPA) was used to decode articulatory gestures in M1, cerebellum and basal ganglia. Furthermore, apart from confirming the role of a mid-M1 region for phonation, we found that a dorsal M1 region, linked to respiratory control, showed significant differences for voiced compared to whispered speech despite matched lung volume observations. This region was also functionally connected to tongue and lip M1 seed regions, underlying its importance in the coordination of speech. Our study confirms and extends current knowledge regarding the neural mechanisms underlying neuromotor speech control, which hold promise to study neural dysfunctions involved in motor-speech disorders non-invasively.

## Introduction

Despite scientific interest in verbal communication, the neural mechanisms supporting speech production remain unclear. The goal of the current study is to capture the underlying representations that support the complex orchestration of articulators, respiration, and phonation needed to produce intelligible speech. Importantly, voiced speech can be defined as an orchestrated task, where concerted phonation-articulation is mediated by respiration^1^. In turn, a more detailed neural specification of these gestures in fluent speakers is necessary to develop biologically plausible models of speech production. The ability to image the speech production circuitry at work using non-invasive methods holds promise for future application in studies that aim to assess potential dysfunction.

Upper motor-neurons located within the primary motor cortex (M1) exhibit a somatotopic organization that projects onto the brain-stem innervating the musculature of speech^2–6^. This functional organization of M1 has been replicated with functional magnetic resonance imaging (fMRI) for the lip, tongue and jaw control regions^7–11^. However, the articulatory control of the velum, which has an active role in natural speech (oral and nasal sounds) remains largely underspecified. Furthermore, laryngeal muscle control, critical for phonation, has more recently been mapped onto two separate areas in M1^4,5,12^: a ventral and a dorsal laryngeal motor area (vLMA and dLMA). Whereas the vLMA (ventral to the tongue motor area) is thought to operate the extrinsic laryngeal muscles, controlling the vertical position of the glottis within the vocal tract, and thereby modulating pitch in voice, the dLMA (dorsal to the lip motor area) is thought to operate intrinsic laryngeal muscles responsible for the adduction and abduction of the vocal cords, which is central to voicing in humans. Isolating the neural control of the intrinsic laryngeal muscles during natural voiced speech is critical for developing a mechanistic understanding of the speaking circuit. At least three research strategies have been adopted in the past in fMRI: (a) contrasting overt (voiced) and covert (imagery) speech^7,11,13^; (b) production of glottal stops^12^; and (c) contrasting voiced and whispered-like (i.e., exhalation) speech^14^. The latter potentially isolates phonation while preserving key naturalistic features of speech, including the sustained and partial adduction of the glottis, the synchronization of phonation, respiration and articulation, and the generation of an acoustic output. In this way, whispered speech can be considered an ecological baseline condition for isolating phonatory processes, free of confounds that may be present with covert speech and the production of glottal stops. Nevertheless, until now, its use has been limited across fMRI studies.

Despite the detailed investigations of M1, the somatotopic organization during overt speech in regions beyond M1 has been relatively unexplored, especially with fMRI. Studying articulatory processes using fMRI has several advantages over other neuroimaging techniques, including high spatial detail^15,16^, and simultaneous cortical and subcortical coverage, which can reveal brain connectivity during speech production helping to achieve a better understanding of the underlying neural circuitry. Despite the benefits of fMRI for speech production research, the signal collected during online speech tasks can be confounded by multiple artefactual sources^17^, for example those associated to head motion^18^ and breathing^19–21^. Head motion is modulated by speech conditions and breathing affects arterial concentrations of CO_2_, regulating cerebral blood flow (CBF) and volume (CBV) and contributing to the measured fMRI signal. Here, we take advantage of several methodological strategies to avoid both head motion and breathing confounds by employing sparse-sampling fMRI^18^ and experimental conditions with well-matched respiratory demands that are measured, respectively.

Using these methods, in this study we investigated fMRI representations of speech production, including articulatory and phonatory gestures across the human cortex and subcortex by employing multivariate decoding methods successfully used in fMRI studies of speech perception^22–24^. Articulatory representations were studied by discriminating individual speech gestures involving the lips, the tongue and the velum. Phonatory representations were studied by contrasting voiced and whispered speech. Furthermore, we recorded lung volume, articulatory measures and speech acoustics to rule out possible non-neural confounds in our analysis. Twenty fluent adults read a list of bi-syllabic non-words, balanced for bilabial and alveolar places of articulation, oral and nasal manners of articulation, and the non-articulated vowel ‘schwa’, using both voiced and whispered speech.

Our analysis employed multivariate decoding, based on anatomically-selected regions of interest (ROIs) that are part of the broad speech production circuitry, in combination with a recursive feature elimination (RFE) strategy^22^. Cortical results were further validated using a searchlight approach that uses a local voxel selection moved across the brain^25^. We expected to find articulatory-specific representations in multiple regions previously linked to somatotopic representations, which included the pre-motor cortex, SMA and pre-SMA, basal-ganglia, brain-stem and cerebellum^26–28^. We further expected to find evidence for larger fMRI responses for voiced in contrast to whispered speech in brain regions implicated in vocal fold adduction (e.g., dLMA^12^). Finally, we investigated functional connectivity using seed regions responsible for lip and tongue control that were possible to localize at the individual subject-level. Accordingly, we expected connections between the different somatotopic organizations across the brain to differ for articulatory and phonatory processes, elucidating the distributed nature of the speaking circuitry^29^ and the systems that support the control of different musculature for fluent speech. Overall, this study aims to replicate and extend prior fMRI work on the neural representations of voiced speech, which includes studying the neuromotor control of key speech articulators and phonation.

## Methods

### Participants

Twenty right-handed participants (5 males), native Spanish speaking, and aged between 20 and 44 years old (mean = 28, sd = 8.14) were recruited to this study using the volunteer recruitment platform (https://www.bcbl.eu/participa) at the Basque Centre on Cognition, Brain and Language (BCBL), Spain. Participation was voluntary and all participants gave their informed consent prior to testing. The experiment was conducted in accordance with the Declaration of Helsinki and approved by the BCBL ethics committee. Participants had MRI experience, and were informed of the scope of the project, and in particular the importance of avoiding head movements during the speech tasks. Two participants were excluded from group analyses due to exceeding head motion in the functional scans. We note that the group sample had an unbalanced number of male and female participants, which should be taken into account when comparing the results of this study to other studies. Attention to gender may be especially important when considering patient populations, where gender seems to play an important role in occurrence/recovery across different speech disorders^30^. Nevertheless, the objective of our research question relates to basic motor skills, which are not expected to differ extensively between male and female healthy adults with comparable levels of fluency^31^. For the voiced speech condition, participants were informed that they should utter the speech sounds at a comfortable low volume level as they would during a conversation with a friend located at one-meter distance. For the whispered speech condition, participants were informed and trained to produce soft whispering, minimizing possible compensatory supra-glottal muscle activation^32^. Because the fMRI sequence employed sparse sampling acquisition that introduced a silent period for production in absence of auditory MR-related noise, participants were trained to synchronize their speech with these silent periods prior to the imaging session, yielding production in more ecological settings.

### Stimuli

Stimuli were composed of written text, presented for 1.5 second in Arial font-style and font-size 40 at the center of the screen with Presentation software (https://www.neurobs.com). Five text items were used (‘bb’, ‘dd’, ‘mm’, ‘nn’ and ‘әә’), where ‘ә’ corresponds to the schwa vowel (V) and consonant-consonant (CC) stimuli were instructed to be pronounced by adding the schwa vowel to form a CVCV utterance (e.g., ‘bb’ was pronounced ‘bәbә’). This assured the same number of letters across the stimuli. The schwa vowel involves minimal or no tongue and lip movements, which promoted a better discrimination of labial from tongue gestures. For the voiced speech task, items were presented in green color (RBG color code = [0 0 1]) and for the whispered speech task in red color (RGB color code = [1 0 0]). Throughout the fMRI acquisitions, we simultaneously obtained auditory recordings of individual (i.e., single token) productions using an MR-compatible microphone (*Optoacoustics, Moshav Mazor, Israel*) placed 2 to 4 cm away from the participants’ mouth. Auditory recordings (sampling rate = 22400 Hz) were used to obtain a list of acoustic measures per token, including speech envelope, spectrogram, formants F1 and F2, and loudness. Loudness was computed based on the average of the absolute acoustic signal in a time window of 100 ms centered at the peak of the speech envelope. Speech envelope was computed using the Hilbert transform: first, an initial high-pass filter was applied to the auditory signal (cut-off frequency = 150 Hz, Butterworth IIR design with filter order 4, implemented with the *filtfilt* Matlab function, Mathworks, version 2014); second, the Hilbert transform was computed using the Matlab function *Hilbert*; finally, the magnitude signal (absolute value) of the Hilbert transform output was low-pass filtered (cut-off frequency = 8 Hz, Butterworth IIR design with filter order 4, implemented with the Matlab function *filtfilt*). The spectrogram was computed using a short-time Fourier transformation based on the Matlab function *spectrogram* with a segment length of 100 time-points, overlap of 90% and 128 frequency intervals. From the spectrogram, F1 and F2 formants were computed based on a linear prediction filter (*lpc* Matlab function).

### Task

**Figure 1:**
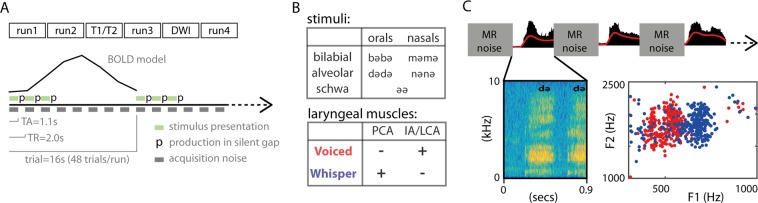
Description of the task. (**A**) Overview of the task: MRI session composed of 4 functional runs divided in trials separated by an inter-trial-interval of 16 s. In each trial, participants produced a given item 3 times. Items are disyllabic non-words (e.g.., bәbә). (**B**) Stimuli and laryngeal control: stimuli was balanced for place of articulation (bilabial and alveolar) and manner of articulation (orals and nasals), and the controlled vowel schwa (ә); for the voiced condition, the IA (interarytenoid) and LCA (lateral cricoarytenoid) laryngeal muscles are recruited, whereas the PCA (posterior cricoarytenoid) is not, and the reversed for the whispered condition. (**C**) Detail of task for a given trial: 0.9 s of silent gap were introduced between consecutive TRs for speech production without MRI noise; top: sound recording in black and low-pass-filtered signal envelope in red; below-left: spectrogram image of an utterance example; below-right: scatter plot of F1 and F2 formants in a given participant (each dot represents an utterance), red for voiced and blue for whispered speech.

The task was to produce a given item either as voiced or whispered speech during a silent gap introduced between consecutive fMRI scans (i.e., sparse sampling). The silent gap was 900 ms. The relevance of speech production during the silent period was three-fold: first, it avoided the Lombard effect (speech intensity compensation due to environmental noise)^33^; second, it limited the contamination of head movements related to speech production during fMRI acquisition^18^; and third, it facilitated voice recording. Trials were presented in a slow event-related design, with an inter-trial-interval (ITI) of 16 seconds. Within each trial, participants read a given item 3 times, separated by a single fMRI volume acquisition (time of repetition, TR = 2000 ms) (Fig. 1A). At each utterance, a written visual text cue was presented for 1500 ms aligned with the beginning of the TR, and as instructed, it indicated participants to utter the corresponding item in the following silent gap (i.e., between 1100 ms and 2000 ms). Item repetition was included to obtain fMRI responses of greater magnitude (i.e., higher contrast-to-noise-ratio, CNR)^34,35^. Between consecutive trials, a fixation cross was presented to maintain the attention of the participants at the center of the visual field. Each run lasted 13 minutes. A session was composed of 4 functional runs. After the second run, two anatomical scans were acquired (T1-weighted and T2-weighted). After the third run, two scans (10 volumes) with opposite phase-encoding directions (anterior-posterior and posterior-anterior) were acquired for in-plane distortion correction. Diffusion weighted imaging (DWI) scans were also acquired between run 3 and 4 for future analyses, but not included in the present analyses.

### MRI acquisition and preprocessing

MRI was acquired at the BCBL facilities using a 3 Tesla Siemens MAGNETOM Prisma-fit scanner with a 64-channel head coil (Erlangen, Germany). Two anatomical scans included a T1-weighted and a T2-weighted MRI-sequences with an isotropic voxel resolution of 1 mm^3^ (176 slices, field of view = 256 × 256 mm, flip angle = 7 degrees; GRAPPA accelaration factor 2). T1-weighted (MPRAGE) used a TR (time of repetition) = 2530 ms and TE (time of echo) = 2.36 ms. T2-weighted (SPACE) used a TR = 3390 ms and TE = 389 ms. These scans were used for anatomical-functional alignment, and for gray-matter segmentation and generation of subject-specific cortical surface reconstructions using FreeSurfer (version 6.0.0, https://surfer.nmr.mgh.harvard.edu). Gray-matter versus white-matter segmentation used the T1-weighted tissue contrast and gray-matter versus cerebral-spinal-fluid (CSF) segmentation used the T2-weighted tissue contrast based on the FreeSurfer segmentation pipeline. Individual segmentations were visually inspected, but none required manual corrections. T2*-weighted functional images were acquired with an isotropic voxel resolution of 2 × 2 × 2 mm^3^ using a gradient-echo (GRE) simultaneous multi-slice (aka multiband) EPI sequence^15,16^ with multiband acceleration factor 5, FOV = 208 × 208 mm (matrix size = 104 × 104), 60 axial slices with no distance factor between slices, flip angle = 78 degrees, TR = 2000 ms including a silent gap of 900 ms, TE = 37 ms, echo spacing = 0.58, bandwidth = 2290 Hz/Px, and anterior-to-posterior (AP) phase-encoding direction. Slices were oriented axially (and in oblique fashion) along the inferior limits of the frontal lobe, brain-stem and cerebellum. In cases where coverage did not guarantee full brain coverage, a portion of the anterior temporal pole was excluded. A delay in TR of 900 ms was introduced between consecutive TRs to allow speech production in absence of MR-related noise and minimize potential head motion artifacts (i.e., TA = 1100 ms). All functional pre-processing steps were performed in AFNI software (version 18.02.16)^36^ using the afni_proc.py program in the individual native space of each participant and included: slice-timing correction; removal of first 3 TRs (i.e., 6 seconds), blip-up (aka, top-up) correction using the AP and PA scans^37^, co-registration of the functional images due to head motion relative to the image with minimal distance from the average displacement; and co-registration between anatomical and functional images.

Simultaneously with fMRI acquisition, physiological signals of respiration (chest volume) and articulation (pressure sensor placed under the chin of the participants) were recorded using the MP150 BIOPAC system (BIOPAC, Goleta, CA, USA). The BIOPAC system included MRI triggers delivered at each TR onset for synchronization between the physiological signals and the fMRI data. The respiratory waveform was measured using an elastic belt placed around the participant’s chest, connected to a silicon-rubber strain assembly (TSD201 module). The belt inputs directly to a respiration amplifier (RSP100C module) at 1000 Hz sampling rate. A low-pass filter with 10 Hz cut-off frequency was applied to the raw respiratory signal. The same sampling rate and low-pass filter was used for the pressure sensor (TSD160C) measuring articulatory movements.

### Univariate analyses

Univariate statistics were based on individual general linear models implemented in AFNI (3dDeconvolve) for each participant. At a first level analysis (subject-level) regressors of interest for each condition type (i.e., 10 condition types: 2 tasks - voiced and whispered, and 5 words - ‘bb’, ‘dd’, ‘mm’, ‘nn’ and ‘ee’) were created using a double-gamma function (*SPMG1*) to model the hemodynamic response function (HRF). Each modelled trial consisted of 3 consecutive production events separated by 1 TR, i.e., 6 second duration. Regressors of non-interest modelling low frequency trends (Legendre polynomials up to order 5) and the 6 realignment parameters (translation and rotation) were included in the design matrix of the GLM. Time points where the Euclidean norm of the derivatives of the realignment motion parameters exceeded 0.4 mm were also included in the GLM to censor occasional excessive motion. At a second level univariate analysis (group-level statistics), volumetric beta value maps were projected onto the cortical surfaces of each subject using SUMA (version Sep. 12 2018, https://afni.nimh.nih.gov/Suma), based on the gray-matter ribbon segmentation obtained in FreeSurfer. Individual cortical surfaces in SUMA were inflated and mapped onto a spherical template based on macro-level curvature (i.e., gyri and sulci landmarks), which guarantees the anatomical alignment across participants. T-tests were employed to obtain group-level statistics on the cortical surfaces using AFNI (*3dttest*++). Statistical maps comprised voiced versus whispered, bilabial versus alveolar, and oral versus nasal items. Group-level alignment of the cerebellum and intra-cerebellar lobules relied on a probabilistic parcelation method^38^ provided in the SUIT toolbox (*version 3.3*, www.diedrichsenlab.org/imaging/suit.htm) in conjunction with SPM (*version 12*, www.fil.ion.ucl.ac.uk/spm) using the T1 and fMRI activation maps in MNI space and NIFTI format. Alignment to a surface-based template of the cerebellum’s gray-matter assured a higher degree of lobule specificity and across subject overlap. All cerebellar maps were projected onto a flat representation of the cerebellum’s gray-matter together with lobule parcelations for display purposes.

Univariate results were not corrected for multiple comparisons. Corrected statistics depended on the sensitivity of MVPA. In order to prepare single trial features for the MVPA analyses (i.e., feature estimation), fMRI responses for each trial and voxel were computed in non-overlapping epochs of 16 secs locked to trial onset (i.e. 9 time points) from the residual pre-processed files after regressing out the Legendre polynomials, realignment parameters and motion censoring volumes. Subsequently, single-trial voxel-wise fMRI responses were demeaned and detrended for a linear slope.

### ROI + RFE MVPA

We adopted an initial MVPA approach based on an anatomical ROI selection using the Desikan-Killiany atlas followed by a nested recursive feature elimination (RFE) procedure^22^ that iteratively selected voxels based on their sensitivity to decode experimental conditions. Thirty-one anatomical ROIs were selected given their predicted role in speech production^3,39^, and covered cortical and sub-cortical regions including the basal-ganglia, cerebellum and brainstem. Because MVPA potentially offers superior sensitivity for discriminating subtle experimental conditions, it was possible to include additional ROIs that have been reported in other human speech production experiments as well as those known to show somatotopy in animal research but that have insofar not shown speech selectivity in human fMRI. The ROIs included the brainstem and a set 15 ROIs per hemisphere: cerebellum (cer), thalamus (thl), caudate (cau), putamen (put), pallidum (pal), hippocampus (hip), pars orbitalis (PrOr), pars opercularis (PrOp), pars triangularis (PrTr), post-central gyrus (ptCG), pre-central gyrus (prCG), supramarginal gyrus (SMG), insula (ins), superior temporal lobe (ST), superior frontal lobe (SF, including SMA - supplementary motor area - and pre-SMA regions). After feature selection, single-trial fMRI estimates were used in multivariate classification using SVM based on a leave-run-out cross-validation procedure. This procedure was used conjointly with RFE^22^. RFE iteratively (here, 10 iterations were used) eliminates the least informative voxels (here, 30% elimination criterion was used) based on a nested cross-validation procedure (here, 40 nested splits based on a 0.9 ratio random selection of trials with replacement was used). In other words, within each cross-validation, SVM classification was applied to the 40 splits iteratively. Every fourth split, we averaged the SVM weights, applied spatial smoothing using a 3D filter [3 × 3 × 3] masked for the current voxel selection and removed the least informative 30% of voxels based on their absolute values. This procedure based on eliminating the least informative features continued for 10 iterations. The final classification accuracy of a given ROI and contrast was computed as the maximum classification obtained across the 10 RFE iterations. Because the maximum criterion is used to obtain sensitivity from the RFE method, chance-level is likely inflated and permutation testing is required. Permutation testing consisted of 100 label permutations, while repeating the same RFE classification procedure for every participant, ROI and classification contrast. This computational procedure is slow but provides sensitivity for detecting spatially distributed multivariate response patterns^23,24^.

Classification for the ROI + RFE procedure was performed using support vector machines (SVM). SVM classification was executed in Matlab using the libsvm library and the sequential minimal optimization (SMO) algorithm. Validation of classification results that is inherent to MVPA was performed using a leave-run-out cross-validation procedure, where one experimental run is left-out for testing, while the data from the remaining runs is used for training the classification model. SVM was performed using a linear kernel for a more direct interpretation of the classification weights obtained during training. Furthermore, fMRI patterns were suggested to reflect somatotopic organizations, thus we expected to observe spatial clustering of voxel preferences in the mapping of the SVM weights. SVM regularization was further used to account for MVPA feature outliers during training, which would otherwise risk overfitting the classification model and reduce model generalization (i.e., produce low classification of the testing set). Regularization in the SMO algorithm is operationalized by the Karush-Kuhn-Tucker (KKT) conditions. We used 5% for KKT, which indicates the ratio of trials allowed to be misclassified during model training. Group-level statistics of the classification accuracies were performed against averaged permutation chance-level using two-tailed t-tests. Multiple comparisons correction (i.e., multiple ROIs) was done using FDR (q < 0.05).

### Searchlight MVPA

In order to further validate the ROI + RFE approach, we conducted a second MVPA approach based on a moving spherical cortical ROI selection^25^. The searchlight allows us to determine whether multivoxel patterns are local. In contrast to the ROI + RFE approach, the searchlight approach is not influenced by the boundaries of the anatomical ROIs. It explores local patterns of fMRI activations by selecting neighboring voxels within a spherical mask (here, 7 mm radius, thus 3 voxels in every direction plus its centroid was used). This spherical selection was moved across the gray matter ribbon of the cortex.

Classification was performed using linear discriminant analysis (LDA)^40^, which allows massive parallel classifications in a short period of time, enabling the statistical validation of the method using label permutations (100 permutations). LDA and SVM have similar classification accuracies when the number of features is relatively low, as it is normally the case in the searchlight method^41^.

Classification validation was based on a leave-run-out cross-validation procedure. Group-level statistics of averaged classification accuracies (across cross-validation splits) were performed against permutation chance-level (theoretical chance-level is 0.5 since all classifications were binary) using two-tailed t-tests. Multiple comparisons correction (i.e., multiple searchlight locations) was done using FDR (q < 0.05). A possible pitfall of volumetric searchlight is that the set of voxels selected in the same searchlight sphere may be close to each other in volumetric distance but far from each other in topographical distance^42^. This issue is particularly problematic when voxels from the frontal lobe and the temporal lobe are considered within the same searchlight sphere. To overcome this possible lack of spatial specificity of the volumetric searchlight analysis, we employed a voxel selection method based on cluster contiguity in Matlab: first, each searchlight sphere was masked by the gray-matter mask; then a 3D clustering analysis was computed (*bwconncomp, with connectivity parameter* = 26); finally, when more than one cluster was found in the masked searchlight selection, voxels from clusters not belonging to the respective centroid cluster were removed from the current searchlight sphere. This assured that voxels from topographically distant portions of the cortical mesh were not mixed.

### Beta time-series functional connectivity

Finally, we explored functional connectivity from seed regions involved in speech articulation. Functional clusters obtained for lip and tongue control in M1 were used because these somatotopic representations were expected to enable localization at the individual subject level. Functional connectivity was assessed using beta time-series correlations^43^. This measure of functional connectivity focuses on the level of fMRI activation summarized per trial and voxel, while neglecting temporal oscillations in the fMRI time-series. Given our relatively slow sampling rate (TR = 2 seconds), this method was chosen over other functional connectivity methods that depend on the fMRI time-series. Pearson correlations were employed to assess the level of synchrony between the average beta time-series of the voxels within each seed region and each brain voxel. This method produces a correlation map (−1 to 1) per seed and participant, converted to z-scores with Fisher’s transformation. Group level statistics were assessed using a two-sided t-test against the null hypothesis of no correlation between the seed regions and brain voxels. Exploring beta time-series correlations in a sub-set of trials from a particular experimental condition relates to the specificity of the functional connectivity measure for that condition independently^43^. Finally, articulatory-specific and phonatory-specific connections were studied using statistical contrasts between the z-scores of different conditions. Hence, articulatory-specific connections of the tongue articulator are those for which the z-scores obtained from tongue-gesture conditions are significantly higher than from lip-gesture conditions, and vice-versa. Phonatory-specific connections of the tongue seed are those for which the z-scores obtained from tongue-voiced conditions are significantly higher than tongue-whispered conditions, and the same for the lip seed.

## Results

### Behavioral and physiological measures

**Figure 2:**
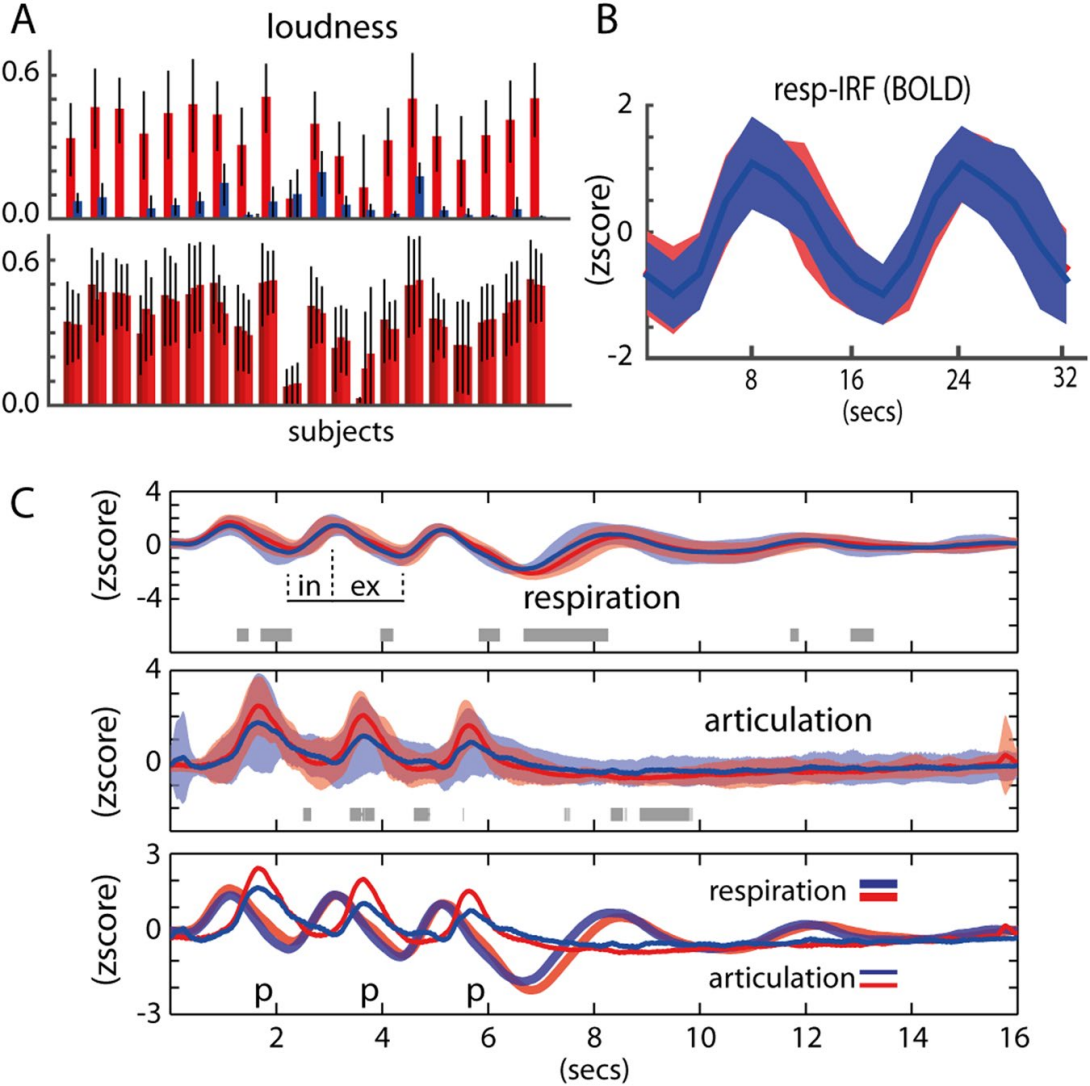
Behavioral results. (**A**) Upper: Loudness per task across all participants. Red is voiced and blue is whispered speech. Bottom: Loudness per item repetition (3 items are produced per trial) across all participants for the voiced speech task. (**B**) Respiratory impulse response function (resp-IRF) using the average BOLD fluctuation within all cortical voxels. (**C**) Group results of the respiratory and articulatory recordings, red is voiced and blue is whispered speech: upper: voiced and whispered respiratory fluctuations (standard errors from the mean is shaded); gray horizontal bars refer to t-test differences (p < 0.05); ‘in’ and ‘ex’ depict inhale and exhale periods, respectively; middle: voiced and whispered articulatory fluctuations; bottom: combined respiratory and articulatory fluctuations.

Chest volume was predictive of speech onset, regardless of the task (i.e., voiced or whispered speech). Measurement of articulatory movements using a pressure sensor placed under the chin of participants was also predictive of speech onset regardless of the speech task (Fig. 2). No significant differences were found between voiced and whispered speech at the group level (FDR q > 0.05) at any point of the averaged time-course of the trials, although in a few time points uncorrected p < 0.05 was found (Fig. 2C, gray shading horizontal bar). Overall, breathing and articulatory patterns were very similar across the production tasks and items. Both at the individual participant level and at the group level, lung volume peak preceded articulation onset. Speech sound recordings obtained synchronously with physiological changes matched the measures of articulatory movements. Speech was successfully synchronized in all participants with our fMRI protocol, i.e., speech was produced within the desired silent periods (900 ms) between consecutive TRs. As expected, voiced speech was significantly louder than whispered speech (p = 8.58 × 10^–10^, Fig. 2A upper panel). The three production events composing a single trial were consistent in loudness, hence variation was small across voiced events (3-way anova, p = 0.99, Fig. 2A lower panel) and the whispered task (p = 0.97). In most participants, the formant F1 extracted from the vowel segments was higher for whispered compared to voiced speech (Fig. 1C) but not for F2. Overall and importantly, we confirmed that the voiced and whispered speech tasks were well-matched for respiration and articulation (Fig. 2C).

We computed an impulse response function (IRF) for the voiced and whispered tasks separately (Fig. 2B). IRF was computed as the averaged fMRI response within all voxels of the cortical gray matter relatively to the onset of trials (hence respiration). It provides a proxy of the respiration impulse response function (resp-IRF) affecting the fMRI signal in the cortex. Due to our experimental design (in particular the stimuli repetition every 16 seconds), the resp-IRF is qualitatively different from the expected respiratory response function in the literature at rest conditions^19^, which commonly shows a long-lasting post-stimulus dip up to around 30 seconds after deep breathing. Here, our post-stimulus dip is interrupted by the onset of the following trial. Despite this qualitative and expected difference, the resp-IRF in this study is marked by an expected peak around 8 seconds after inhalation. Importantly, no differences in the shape of the resp-IRF were found between the voiced and whispered tasks, making our design proof to variation in brain oxygenation related with respiratory behavior.

### Univariate

**Figure 3:**
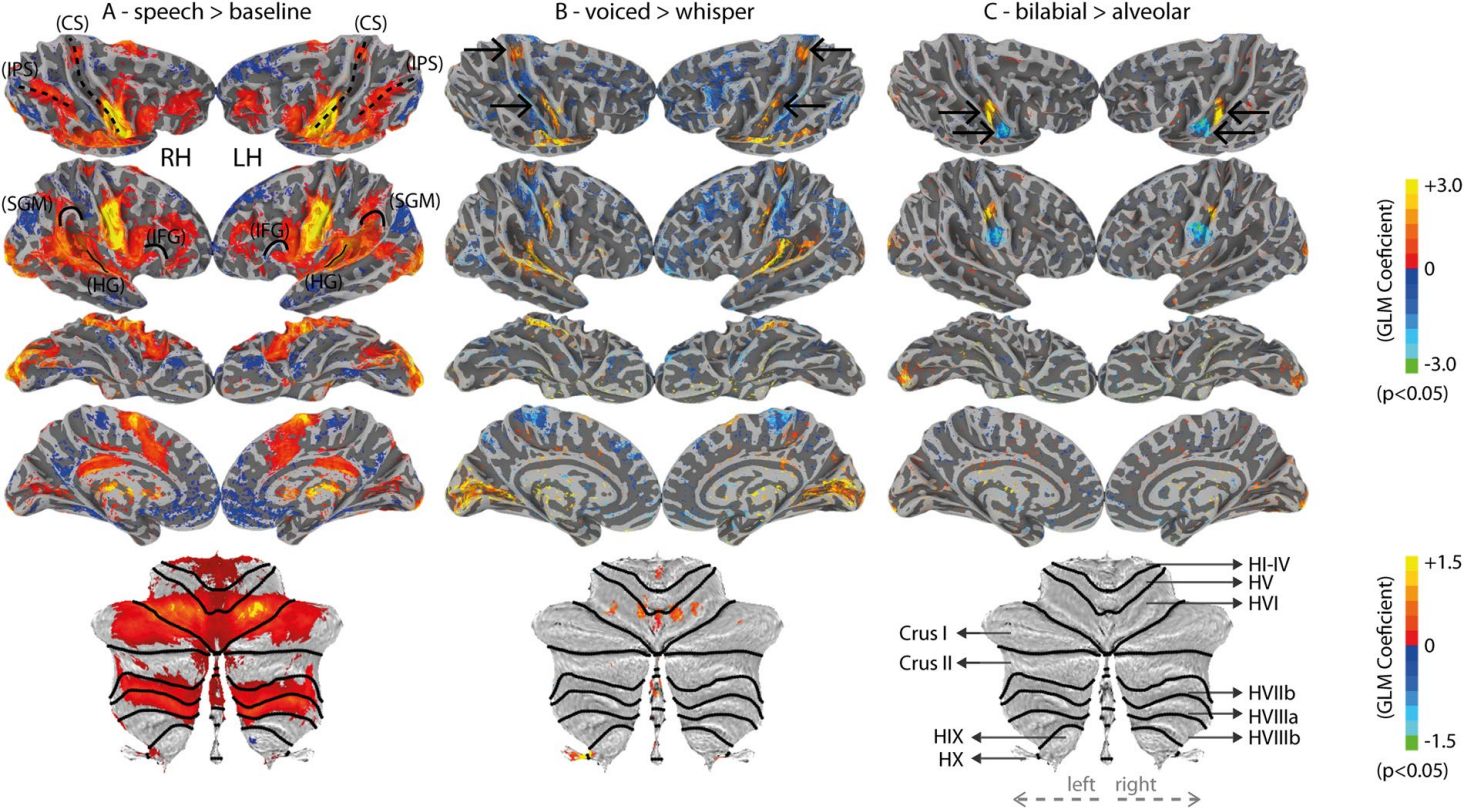
Univariate fMRI results (uncorrected statistics). (**A**) Speech versus baseline. The central sulcus (CS), inferior parietal sulcus (IPS), supramarginal gyrus (SMG), Heschl’s gyrus (HG) and inferior frontal gyrus (IFG) are outlined to provide landmark references. Bottom: flat cerebellum map; black lines represent borders between cerebellar lobules. (**B**) Voiced versus whispered speech. Top arrows indicate the trunk motor area (TMA) and the bottom arrows indicate the dorsal laryngeal motor area (dLMA) found by this contrast. (**C**) Bilabial versus Alveolar conditions. Top arrows indicate the lip and the bottom arrows the tongue motor regions. Bottom cerebellum representation includes labels of the parcelated cerebellar lobules according to the SUIT atlas^42^.

Producing speech relied on the typical brain network for reading and speaking, regardless of task (speech > baseline, Fig. 3A). This network was observed bilaterally, and included areas of the occipital cortex, the intra-parietal-sulcus (IPS), the auditory cortex and posterior medial temporal gyrus (MTG), the ventral motor cortex and ventral pre-motor cortex, the superior medial frontal cortex (including the supplementary motor area, SMA, and pre-SMA), the inferior frontal gyrus, a superior portion of the lateral motor-cortex (central-sulcus), the supramarginal gyrus, the anterior insula, the posterior cingulate, and also extended to areas of the cerebellum (including the lobule HVI, Crus I, lobule HVIIb and HVIIIa) and basal ganglia. It is important to note that occipital cortex effects were expected given that the cues for the utterances were visually presented (in orthographic form) (Fig. 3A). These visually-based fMRI activations may be also present for the voiced vs. whispered speech contrast due to differences in the color of the stimuli and attention therein (Fig. 3B). Other contrasts within the speech task conditions may also elicit occipital effects due to potential differences in brain orthographic representations, including the ventral occipitotemporal cortex.

In comparison to whispered speech, voiced speech yielded stronger univariate responses in the auditory regions, the dorsal central sulci (dorsal-M1), some portions of middle central sulci (mid-M1), and the lobule HVI of the cerebellum (Fig. 3B). Conversely, whispered speech showed stronger fMRI responses within the posterior bank of the post-central gyrus (somatosensory cortex) bilaterally and distributed portions of the frontal lobes (Fig. 3B). Compatible to our expectations, ventral M1 showed a somatotopic organization for lip and tongue items along the superior-inferior direction, respectively (Fig. 3C). This organization was marked (p < 0.05) in 11 out of the 18 participants, as well as, at the group level. No significant differences were found between oral and nasal utterances in the univariate analysis.

### ROI + RFE MVPA

**Figure 4:**
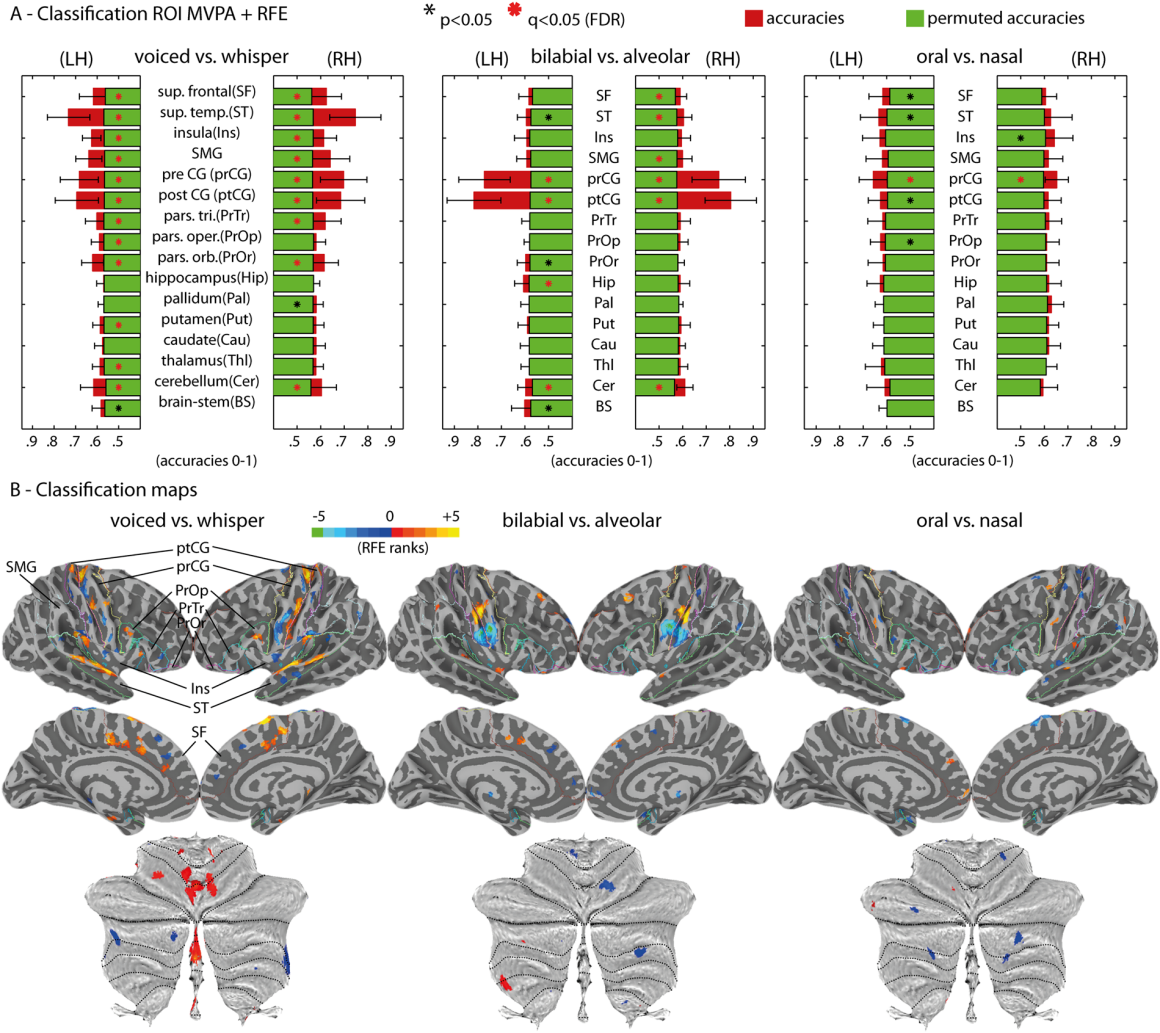
Multivariate fMRI group results. (**A**) MVPA ROI + RFE results for the three main contrasts: voiced versus whispered speech; bilabial versus alveolar; oral versus nasal. Classification is depicted by red bars and permutation chance-level by green bars. Black-colored asterisks (*) represent two-sided paired t-test of classification results against permutation chance-level (p < 0.05); red-colored asterisks represent FDR corrected statistics (q < 0.05) for multiple ROI tests. (**B**) Classification importance of the voxels within each ROI (using the RFE algorithm) projected onto the cortical and cerebellar maps. Multiple ROIs are projected simultaneously onto the maps for simplicity; the boundaries of the ROIs are indicated as colored lines and labelled in the top-left map. The sign of the voxel’s importance (positive or negative) represent their preference towards the first condition (positive values, warm colors) or second condition (negative values, cold colors). Bottom: maps of voxel’s importance in the cerebellum.

**Figure 5:**
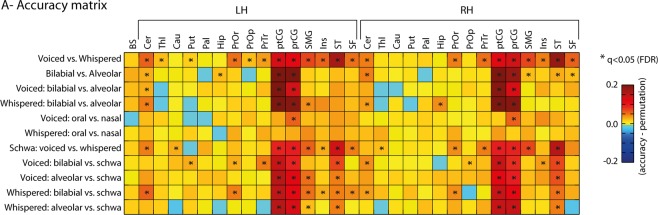
Summary of MVPA ROI + RFE results. Matrix reporting the accuracy difference between classification and permutation chance-level. Asterisks (*) indicate significant FDR q < 0.05. ROI results are shown for the brainstem, left ROIs and right ROIs. ROI labels are: BS (brain stem); Cer (cerebellum); Thl (thalamus); Cau (caudate); Put (putamen); Pal (pallidum); Hip (hippocampus); PrOr (pars orbitalis); PrOp (pars opercularis); PrTr (pars triangularis); ptCG (post-central gyrus); prCG (pre-central gyrus); SMG (supramarginal gyrus); Ins (insula); ST (superior temporal); SF (superior frontal).

Decoding was independently conducted per ROI to unravel fMRI representations of articulatory and phonatory processes during speech production (Fig. 4). This strategy revealed decoding classifications significantly above permutation chance level in multiple ROIs, cortically and sub-cortically, across the experimental contrasts. Specifically, in voiced versus whispered speech conditions, we found significantly higher classification (q < 0.05) on most of the cortical ROIs except the right pars opercularis (PrOr), and in subcortical ROIs, including the cerebellum bilaterally, left thalamus (Thl) and left putamen (Put). Classification of articulatory differences based on lip versus tongue gestures (‘bb’ + ‘mm’ versus ‘dd’ + ‘nn’) regardless of task was significant (q < 0.05) in the pre-central (prCG) and post-central gyri (ptCG), cerebellum bilaterally, right superior frontal (SF), right superior temporal (ST), and left hippocampus (Hip). Classification of oral versus nasal gestures in the voiced speech task revealed the exclusive involvement of the prCG bilaterally (q < 0.05), but of no other ROI. Nasality was investigated strictly in the voiced speech task due to possible uninterpretable differences of velum control during the whispered speech task^32^. Importantly, the left putamen showed significant (q < 0.05) classification of bilabial versus the schwa vowel in the voiced speech tasks. Maps of RFE voxel selection (Fig. 4) for all ROIs are depicted conjointly in the cortical surfaces. The sign of the RFE maps indicates preference towards the first class (positive values) versus second class (negative values). RFE maps correspond with the uncorrected univariate statistics depicted in Fig. 3. Furthermore, additional classification contrasts targeted the schwa vowel conditions (see Fig. 5 for a complete set of classification contrasts).

### Searchlight MVPA

**Figure 6:**
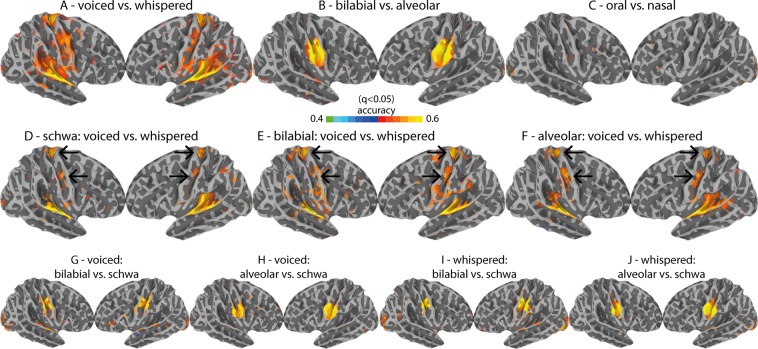
Cortical searchlight results. (**A–C**) the main contrasts (voiced versus whispered, bilabial versus alveolar, oral versus nasal). (**D–F**) task-based contrasts (voiced versus whispered speech) done separately by stimuli type (schwa, bilabial and alveolar). Top arrows indicate the consistency of TMA location; Bottom arrows indicate the consistency of LMA location. Arrows are placed equally on every map. (**G–J**) contrasts of each place of articulatory condition (bilabial and alveolar) versus the schwa condition for each task separately (voiced and whispered speech).

The searchlight analysis (Fig. 6), which is not restricted to pre-defined anatomical boundaries (i.e., anatomical ROIs), was used as a complementary method for cortical classification output. This additional classification strategy allows disentangling whether representational fMRI patterns are distributed within the cortical ROIs or instead focal, as investigated using the searchlight approach. The searchlight results were consistent with the ROI-based MVPA results, with the exception of the contrast for oral versus nasal gestures. Oral vs. nasal gestures were successfully discriminated with the ROI-based method but not with the searchlight-based method. All searchlight maps were corrected for multiple searchlight comparisons using FDR (q < 0.05). The effect of phonation (voiced versus whispered speech) across multiple speech items (i.e., schwa, bilabial and alveolar, see Fig. 6) validated the role of middle and dorsal M1 regions (see black arrows), in addition to the involvement of the temporal lobe (superior temporal gyrus, STG) during voiced speech.

### Functional connectivity

**Figure 7:**
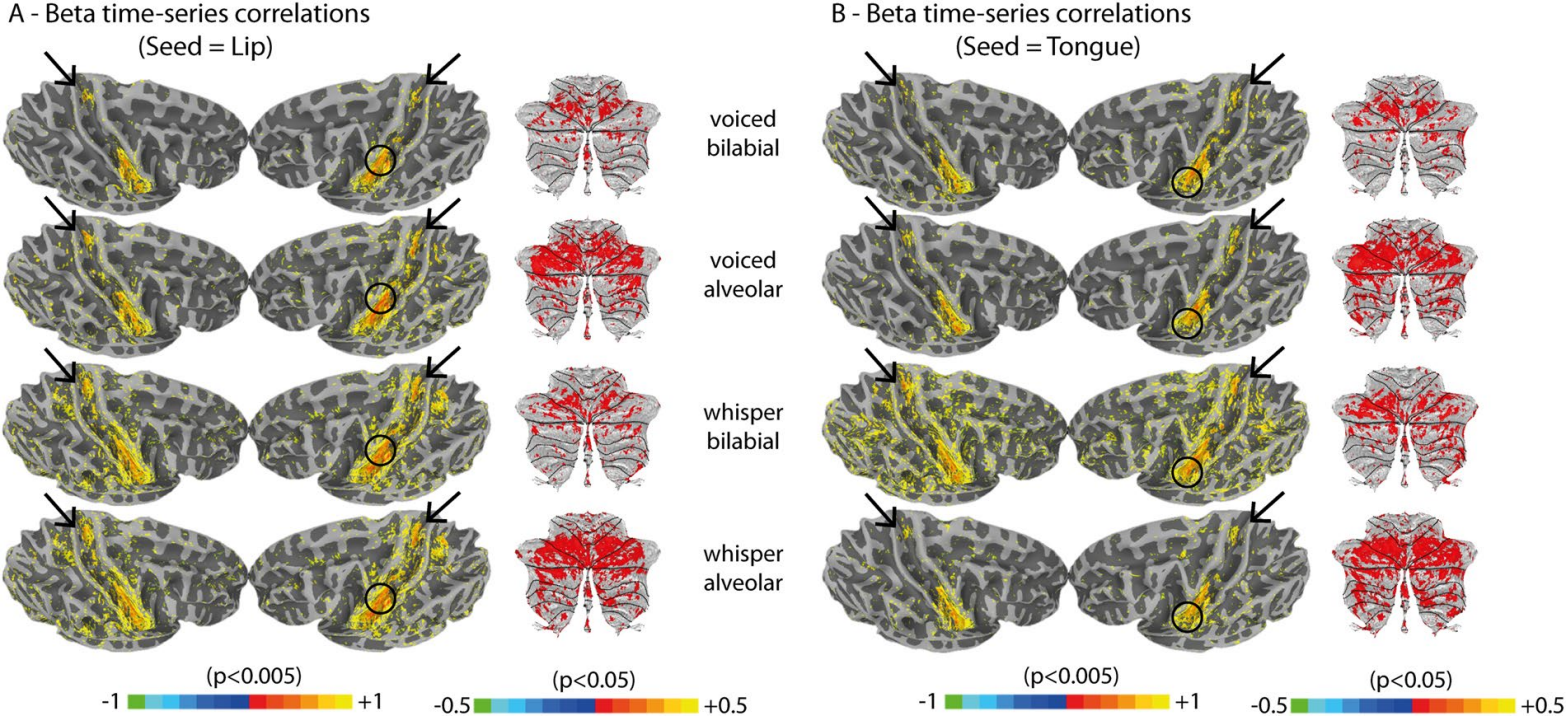
Functional connectivity results using beta-time-series correlations based on individually localized lip and tongue motor regions. Circles indicate an approximate localization of the seed region in the group-inflated surface (seed regions were identified at the individual subject level using a p < 0.05 threshold). Black arrows highlight the location of the trunk motor area (TMA). (**A**) Seed is the left lip motor region. (**B**) Seed is the left tongue motor region.

Beta time-series correlations (Fig. 7) were performed to explore functional connectivity using M1 seed regions individually identified (i.e., lip and tongue somatotopy during our task). Overall, correlations were similar across the seed regions, despite the different speech conditions (i.e., different items and tasks). Beyond voxels contiguous to the seed regions, we found that the dorsal M1 region (suggested to be involved in respiration and therefore phonation) is significantly correlated with the ventral articulatory seed regions for all speech conditions (p < 0.005). Furthermore, the cerebellum also showed correlations with the seed regions, albeit less pronounced (p < 0.05), especially in regions within the lobule HVI, bilaterally. Together, dorsal M1 and the cerebellum were the only brain regions non-contiguous to the seed regions that showed consistent clustered correlations. Because correlations were also performed within each speech task and articulatory gesture type, separately, they are immune to fMRI signal modulations across these conditions. Finally, we did not find articulatory-specific nor phonatory-specific connectivity. We tested whether a preference of task (voiced or whispered speech) or place of articulation (bilabial and alveolar) existed in the connectivity maps for the lip and tongue regions separately. None was found. Furthermore, we tested whether a preference of left versus right seed regions existed in connectivity maps, but this was not the case (all subjects were right-handed).

## Discussion

**Figure 8:**
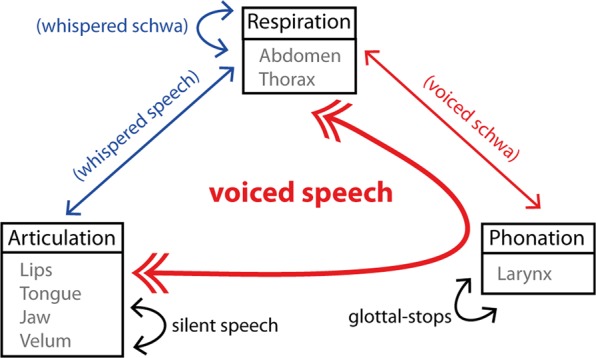
Summary diagram of the cooperation required for voiced speech and possible experimental conditions used to isolate the sub-components of speech production (Respiration, Phonation and Articulation).

Speech production involves the concerted control of multiple neuromotor systems, including articulation, phonation and respiration (Fig. 8). The current study contributes to the existing literature by capitalizing on MVPA methods with high-spatial resolution fMRI, providing a non-invasive tool, sensitive enough to uncover articulatory-specific representations within and beyond M1. These findings are promising for investigating the speech production circuitry in humans and *in-vivo*, and to investigate the dysfunctions that can be potentially present in neuromotor speech disorders.

Although primary motor regions (M1) hold somatotopically-organized neural connections to the human musculature, including those required for speaking, a number of other cortical and subcortical regions are also suggested to be of critical importance^26,29,44^. Mapping articulatory speech gestures, involving the lips, tongue and velum, only solves one piece of the complex articulatory control puzzle. Another important piece of the puzzle is how these gestures occur in concert with phonation, and consequently respiration. The orchestration of these three systems is unique and central to verbal communication in humans and must be studied in such a way that preserves important ecological features of intelligible speech, including the sustained and partial adduction of the glottis, and the synchronization of phonation, respiration and articulation. To this end, the current fMRI study investigated the representations involved in the production of lip, tongue and velum gestures, as well as the larynx (phonation). In addition to univariate analyses, MVPA decoding strategies^22–25^ were employed. These methods proved essential in revealing the subtle articulatory-specific fMRI response patterns in a group of fluent adults, namely in subcortical regions, and between oral and nasal items. In addition, this study took advantage of simultaneous multi-slice acquisition in order to sample the signal every 2 seconds, including a necessary 900 ms gap for speaking in absence of MR acquisition that has been shown to mitigate the confounding effects of head motion^18^. Nevertheless, despite the silent acquisition paradigm, occasionally participants’ productions overlapped with the scanner noise of the subsequent TR. This unexpected occasional overlap can affect the online monitoring of re-afferent speech^45^, concerning the vowel segment but not the consonant segment, which is most critical in our analyses of articulatory (place of articulation of the consonants) and phonatory mechanisms of speech production.

The results implicate several brain regions in the neuromotor control of articulation (i.e., bilabial versus alveolar and oral versus nasal) and phonation (i.e., voiced versus whispered speech). Specifically, we observed distinct superior and middle subregions of M1 that showed significantly greater fMRI responses for voiced compared to whispered speech. The finding in the mid M1 region is consistent with prior work showing its involvement in controlling intrinsic laryngeal muscles during glottal stops, in humans (a.k.a., dLMA)^7,8,46^. For the superior M1 region, which is thought to be responsible for the voluntary control of breathing (a.k.a., trunk motor area, TMA)^4,8,10,14^, our results suggest that beyond managing air-flow, it is particularly involved in phonation. Although the use of whispered speech have previously been studied using positron emission tomography (PET)^47^ and lower spatial resolution fMRI^14^, the high spatial resolution fMRI, in combination with sparse-sampling, well-matched respiratory conditions and MVPA employed here allowed us to further specify differences between these conditions. Beyond cerebral cortex, we identified phonatory and articulatory representations in superior portions of the cerebellum (lobule HVI), whose predicted involvement in the timely management of motor execution and integration of online somatosensory and auditory feedback^48^ make it a key contributor to the production of speech^39,49^.

Despite the advantage of using whispered speech as a baseline condition for isolating phonation, while controlling for respiratory behavior, it may involve compensatory gestures needed for producing intelligible whispered speech. In fact, the sound effect present in whispering results from supra-glottal laryngeal muscles (i.e., tyropharyngeus) that constrain the passage of air downstream from the glottis^32^. To minimize potential effects related to compensatory supra-glottal muscles in our study, we trained participants to produce ‘soft’ whispered speech, which has been shown to significantly eliminate electrophysiological activations in these muscles^32^. Furthermore, both voiced and whispered speech depend on expiratory air-pressure. Importantly, our chest volume recordings did not show differences between voiced and whispered speech, suggesting that our voicing contrast was not confounded by respiration^20,47^.

Ventral M1, the cortical primary motor area for the control of articulated speech, has received increased attention in the past decades by fMRI^9,12^ and intracranical EEG^5,50,51^ research. Within ventral M1, we found dissociation for place of articulation (lip versus tongue) and nasality (nasals versus orals), confirming distinct somatotopic evidence^4,5,7,10^. Beyond M1, representation of place of articulation was found in multivoxel patterns of the left and right cerebellum and the right superior temporal lobe (Fig. 4). We also found articulatory representations in the left putamen in the basal ganglia, although these were restricted to the comparison of bilabial gestures versus the ‘schwa’ vowel (Fig. 5). Cortico-striatum-cortical loops are critical for neuromotor control. However, detailed representations and connections in the basal ganglia during speech tasks remain unclear. It is unknown whether communication between M1 and the basal ganglia depend on parallel connections (i.e., somatotopically preserved connections), or whether neural convergence occurs in the circuit. Recent animal electrophysiology^26^ suggests that a certain level of convergence takes place in the cortico-striatum-cortical loop, which explains lack of articulatory-specific representations found at the basal ganglia in this study. Alternatively, identifying articulatory-specific representations in specific basal ganglia nuclei may require higher spatial resolution. Here, 2 mm isotropic voxels in 3-tesla MRI were used. In the future, imaging the basal ganglia at higher magnetic field strengths (e.g., 7-tesla MRI) may render the necessary resolution to study speech representations within these brain structures typically suffering from reduced fMRI signal^52^.

With respect to the findings in the cerebellum, several recent neuro-anatomical accounts of speech production incorporate cortico-cerebellar-cortical interactions for the coordination of time-dependent motor planning, monitoring and execution^48,53,54^. Indeed, we found both articulatory and phonatory representations in the cerebellum, in particular within lobule HVI, bilaterally. Importantly, the identified cerebellar regions were functionally connected to ventral M1 (Fig. 7). Previous research has shown somatotopic representations of tongue movements in Lobule HVI in the absence of speech^38,55^. Here, we were able to specify the spatial location of lip and tongue representations, as well as a separate locus for the control of the larynx, in lobule HVI; laryngeal representations were more medial compared to lips and tongue (Fig. 4B).

To our knowledge, no fMRI studies have examined the representation of velum control. Velum control includes the action of several/distributed muscles^56^, which limits its topographical localization. The results of the current study show that the classification of oral versus nasal sounds (i.e., velum control) using MVPA was significant within the pre-central gyri, bilaterally (Figs. 4 and 5). Interestingly, focal brain regions representing velum control were not found in our searchlight analysis. Taken together, our results suggest a more distributed representation of the velum control across M1. In future, a more deterministic understanding of the somatotopy of the velum may require investigating velar movements separately, for example by using continuous oral to nasal (and nasal to oral) vowel transitions, or by simultaneously obtaining the velum’s position using real-time MRI^57,58^ or non-invasive electromyogram recordings^56^.

We also probed the potential specificity of functional connections for lip vs. tongue articulatory and voiced vs. whispered speech to better characterize the coordination of the neural circuitry involved in speech. Somatotopically-based connections have been unveiled for left- and right-hand movements between M1 and the cerebellum in human fMRI^55^ and to some extent between M1 and the basal ganglia in animal electrophysiology^26^. Notwithstanding, it remains unclear whether the specificity of connections are preserved across brain regions involved in their motor control (i.e., parallel connections) or instead show convergence^26^. In this study, articulatory-specific or phonatory-specific functional connections were not found, supporting convergence of functional connectivity for the speech gestures here studied and during online speech production. It is however possible that faster fMRI acquisition rates (e.g., below 1 second) in combination with functional connectivity methods that rely on fMRI time-series (e.g., psychophysiological interactions or dynamic causal modelling) would allow us to unravel specificity of neural connections during speech production.

In conclusion, we investigated the brain’s representation for speech production using whole-brain fMRI coverage. We applied univariate and multivariate decoding analyses to define articulatory representations involving the lips, tongue and velum, as well as representations of phonation using voiced and whispered speech. Our results confirmed the role of a region within the mid portion of the central sulcus (dLMA) for voiced speech^12,14,46^. Importantly, we found that voiced speech additionally recruits a superior motor area, previously implicated in controlling trunk respiratory muscles^4^. Since our task had matched respiratory behavior, this trunk motor region does not seem to reflect differences in respiratory control, but instead to subserve the coordination between articulation, respiration and phonation. This interpretation is further supported by the fact that this region was functionally connected to tongue and lip M1 subregions. Our results also indicate that the cerebellum spatially dissociates articulatory and phonatory gestures, supporting an extensive literature on the role of the cerebellum, both as a motor mediator and a processing unit for sensory feedback^48^. Moreover, multivariate decoding showed evidence for the active role of the putamen in phonation and articulation. It is assumed that somatotopically-preserved connections are part of cortico-striatum-cortical loops^26^, and debate remains on whether these circuits are recruited in the linguistically-matured brain, or specific to the development of the motor skills involved in speaking^59^. Taken together, the results of the current study suggest that MVPA provide an effective method for investigating speech representations during naturalistic conditions using non-invasive brain imaging technology. Thus, they can be used to better understand the neurobiology of speech production both in fluent and disordered populations, conduct longitudinal studies reflecting speech development, as well as assess the benefits of clinical interventions.
